# Supplementary material for: Content-rich biological network constructed by mining PubMed abstracts
Source: BMC Bioinformatics. 2004 Oct 8;5:147. doi: 10.1186/1471-2105-5-147 (PMC528731; doi:10.1186/1471-2105-5-147)
Supplement: Additional File 2 — The original results of the above study (non-essential files are deleted to keep the file size under the limit set by BMC bioinformatics). [file 1471-2105-5-147-S2.bz2 › chilibotAdditionalFile2/dip30/433ID7622448E10378/html/CCL3_CCR3.html]

 


 **CCL3** and **CCR3** 
  
Found 21 abstracts in PubMed, retrieved 21.  
 

 What does Google say? 
 PDF only 
| .edu only 

---

**Interactive relationship** (e.g. stimulation, inhibition, etc)

**Neutral relationship**- Here we show that the mouse orphan receptor previously named macrophage inflammatory protein 1 alpha  [ **CCL3** ]  receptor like 2 is a functional eotaxin receptor  [ **CCR3** ] .  Ref: 8687456 Biochem Biophys Res Commun, 1996
- The other murine beta chemokine receptor, which we have designated murine CCR 3  [ **CCR3** ] , also binds murine MIP 1 alpha  [ **CCL3** ] .  Ref: 7594543 J Immunol, 1995
- We and others have shown that the human beta chemokine  **CCL3**  L1, unlike its related non allelic isoform  **CCL3** , has high affinity for the chemokine receptors D6,  **CCR3**  and CCR5.  Ref: 12355456 Eur J Immunol, 2002
- When Ca2 i changes were monitored in stably transfected human embryonic kidney 293 cells, MIP 1 alpha  [ **CCL3** ]  and RANTES were both potent agonists for CC CKR3  [ **CCR3** ]  and CC CKR1.  Ref: 7622448 J Biol Chem, 1995
- Contrary to LD78alpha  [ **CCL3** ] , LD78beta 1 70 at 30 ng ml efficiently competed with 125I RANTES for binding to  **CCR3**  and mobilized calcium in  **CCR3**  transfectants.  Ref: 11449371 Eur J Immunol, 2001
- CC CKR3  [ **CCR3** ]  is a seven transmembrane domain G protein coupled receptor most closely related to the previously reported monocyte and neutrophil selective receptor CC CKR1 also known as the MIP 1 alpha  [ **CCL3** ]  RANTES receptor.  Ref: 7622448 J Biol Chem, 1995
- However, human neutrophils do not migrate when the cells are stimulated with other human CC chemokines, such as human macrophage inflammatory protein 1 alpha  [ **CCL3** ]  hMIP 1 alpha and eotaxin, which also use the CCR1 and  **CCR3**  as their receptors.  Ref: 10202040 J Immunol, 1999

**Non-interactive relationship** (e.g. studied together, co-existance, homology, etc.)

- The most active chemokines for resting Th IL 10, in terms of calcium mobilization and in vitro migration, were in order of potency CCL2 monocyte chemoattractant protein 1, CCR2 ligand, CCL4 macrophage inflammatory protein 1beta, CCR5 ligand,  **CCL3**  macrophage inflammatory protein 1alpha, CCR1 5 ligand, CCL17 thymus and activation regulated chemokine, CCR4 ligand, CCL1 I 309, CCR8 ligand, CXCL12 stromal derived factor 1, CXCR4, and CCL11 eotaxin,  **CCR3**  ligand.  Ref: 11145678 J Immunol, 2001
- CC CKR3  [ **CCR3** ]  also bound MCP 1 with lower affinity, but did not bind MIP 1 alpha  [ **CCL3** ]  or MIP 1 beta.  Ref: 8642344 J Exp Med, 1996
- Using self organizing maps, we identified nine genes CD4,  **CCR3** , CCR5, LT beta, MIP 1 alpha  [ **CCL3** ] , MIP 2, CD8 alpha, IP 10, and RANTES, each with a unique profile of transcriptional expression, that reproduce the differentiation of states of rejection in dendrograms.  Ref: 11818457 J Leukoc Biol, 2002
